# Effect of air filtration on house dust mite, cat and dog allergens and particulate matter in homes

**DOI:** 10.1002/clt2.12137

**Published:** 2022-04-21

**Authors:** José María Maya‐Manzano, Gudrun Pusch, Cordula Ebner von Eschenbach, Elke Bartusel, Thomas Belzner, Erwin Karg, Ulrich Bardolatzy, Michael Scheja, Carsten Schmidt‐Weber, Jeroen Buters

**Affiliations:** ^1^ Center of Allergy & Environment (ZAUM) Member of the German Center for Lung Research (DZL) Technical University and Helmholtz Center Munich Germany; ^2^ Philips Research China Shanghai China

**Keywords:** air filtration, HEPA, indoor allergens, particulate matter, UFP

## Abstract

**Background:**

Indoor allergens (i.e. from mite, cat and dog) are carried by airborne particulate matter. Thus, removal of particles would reduce allergen exposure. This work aims to assess the performance of air filtration on particulate matter and thus allergen removal in 22 bedrooms.

**Methods:**

Indoor air was sampled (with and without air filtration) with a cascade impactor and allergens were measured using enzyme‐linked immunosorbent assay (ELISA). Particulate matter (including ultrafine particles) was also monitored.

**Results:**

The median of allergen reduction was 75.2% for Der f 1 (*p* < 0.001, *n* = 20), 65.5% for Der p 1 (*p* = 0.066, *n* = 4), 76.6% for Fel d 1 (*p* < 0.01, *n* = 21) and 89.3% for Can f 1 (*p* < 0.01, *n* = 10). For size fractions, reductions were statistically significant for Der f 1 (all *p* < 0.001), Can f 1 (PM_>10_ and PM_2.5–10_, *p* < 0.01) and Fel d 1 (PM_2.5–10_, *p* < 0.01), but not for Der p 1 (all *p* > 0.05). PM was reduced in all fractions (*p* < 0.001). The allergens were found in all particle size fractions, higher mite allergens in the PM_>10_ and for pet allergens in the PM_2.5–10_.

**Conclusions:**

Air filtration was effective in removing mites, cat and dog allergens and also particulate matter from ambient indoor air, offering a fast and simple solution to mitigate allergen exposome.

## 1 INTRODUCTION

Indoor allergen exposure has a substantial negative impact on the quality of life of allergic rhinitis patients,^1^ and house dust mite (HDM's) allergens are the most important indoor allergens,^2^, ^3^, ^4^ especially Der p 1, Der p 2 and Der p 23 from *Dermatophagoides pteronyssinus* ^5^, ^6^ and Der f 1 and Der f 2 from *Dermatophagoides farinae*.^7^, ^8^ Mites' allergy shows a sensitization rate in Germany of 23.5% for *D. pteronyssinus* and 21.1% for *D. farinae*.^9^ HDM allergens are mostly (95%) from mite's faecal pellets^4^, ^10^ and were detected in the fraction >10 μm that becomes airborne after being disturbed.^11^, ^12^, ^13^ Still, around one fifth of total airborne mite allergen was found to be carried by particles <4.7 μm,^13^ and authors reported the possibility of larger particles becoming fragmented.^14^ Settling velocity increases with increasing aerodynamic diameter of a particle. Thus both, the smaller particles with capacity of penetrating deeper into the airways^5^, ^15^ and the larger particles containing the majority of the allergen^16^ should be removed.^13^, ^16^, ^17^, ^18^

Der f 1 has a half‐life of 10 years^8^, ^19^ and it is not realistic to rely on natural decay to ensure low allergen levels at homes. The bundle of single measures recommended to mitigate mite allergen exposure (encasings, washing bedding >60°C, humidity <50%, removal of carpets etc.) is quite diverse,^4^, ^20^, ^21^, ^22^, ^23^, ^24^, ^25^ but a combination of them is typically advocated,^26^, ^27^, ^28^ as the ‘one only’ approach was often reported as ineffective.^1^, ^4^

Other indoor allergens such as those originating from cats and dogs can also be ubiquitous, even when these animals are absent in the homes.^29^, ^30^ Fel d 1 is the major cat allergen,^31^ originating from the sebaceous gland of the skin,^32^ whilst Can f one is found mostly in saliva and hair/dander.^33^ Removing the pet from the household is considered effective for allergen avoidance, but most pet owners are not willing to do so.^34^

Since these allergens are detected in the airborne PM, a good approach to reduce exposure would be filtering the air.^21^, ^35^, ^36^ In the last few years, significant improvements in allergic rhinitis and asthma symptoms were reported using high‐efficiency particulate air (HEPA) air purifiers,^37^, ^38^ which also resulted in a decrease in medication.^38^ HEPA air purifiers not only removed the HDM allergens,^39^, ^40^ but also cat allergens.^41^ Besides exposure to allergens, there are other concerns about exposure to PM. The health effects of PM, especially PM_2.5_, are well described and include respiratory and cardiovascular diseases, lung cancer and cognitive dysfunctions,^42^, ^43^ especially in vulnerable groups like children, elderly and people with pre‐existing cardiovascular diseases.^42^, ^43^ Ultrafine particles (UFP) exposure is a concern too, and evidence suggests adverse effects on cardiovascular and cerebrovascular health.^44^, ^45^ Thus, although the effectiveness of air filtration has been proven for certain particle sizes,^38^, ^39^ the novelty of our study is to simultaneously investigate the airborne levels of three major indoor allergens in different size fractions as well as PM covering a wide range of sizes in homes (bedrooms).

The aim was to determine exposure to airborne allergens from HDM, cats and dogs, PM and UFP in 22 bedrooms in Bavaria, South Germany.

Then we tested whether these parameters can be significantly reduced by using air filtration, for which we used a portable air purifier (Philips Air Purifier AC4236, 4000i‐series) with a HEPA filter and a clean air delivery rate (CADR) of 500 m^3^/h.

## 2 MATERIAL AND METHODS

### 2.1 Recruitment process to select homes

Approvals from the ethical committee of Klinikum rechts der Isar, number 377/19‐S‐SR, amendment from 23 July 2020 (SARS‐CoV2 measures) and from Philips Internal Committee Biomedical Experiments were obtained. Twenty‐two homes were selected according to the following inclusion/exclusion criteria and informed consent was obtained in writing.

### 2.2 Inclusion criteria

- All occupants being between 18 and 65 years of age.
- homes within a radius of about 50 km of Munich, preferentially detached or semi‐detached.
- the bedroom needed to have a door separating it from other rooms.
- Bedroom at ground or first floor.
- Age of mattress >4 years.
- Willing not to change their bedding for 2 weeks before each home visits.
- Not traveling for more than 1 week during the study duration (i.e. 5 weeks).
- Preferred people sleeping in winter ‘always’ with closed windows over ‘sometimes’, over ‘never’.
- Fluent German and/or English speaking ‘willing and able to provide informed consent’.

### 2.3 Exclusion criteria

- Unwilling or unable to provide informed consent.
- Being absent from their homes for more than 1 week during the duration of the study.
- Households where at least one occupant had asthma.
- Bedroom height more than 3.2 m.
- Bunk beds and water beds in bed room.
- Mattress cleaned by vacuuming within previous 6 months.
- Air brick or ventilation aperture/mechanical ventilation in the bedroom.
- Households using air dehumidifiers in their homes.
- Households using house dust mite kill sprays/products.
- Households using mattress encasings in bedroom.

### 2.4 Experimental design and equipment

The study period was from 5 February 2020 to 22 April 2020 and (after a break due to a SARS‐CoV‐2 lockdown in Germany) from 1 July 2020 to 28 September 2020 (Table S1). Every home completed a control‐ and intervention visit following a crossover randomized experimental design, having both visits within 4 weeks to avoid any influence or bias provoked by the possible seasonality of mites or pet allergens. Thus, half of cases were first control (without air filtration) and half first intervention (when air filtration was working during sampling).

To collect airborne particles a *GMU* (Gerhard Mercator Universität, Duisburg) *Johnas II* cascade impactor (thereafter Johnas 2) was used, connected to a pump with an aspiration rate of 53 l/min. Flow was permanently monitored in‐line with a *Bellows BG series* gas flow meter. The flow was additionally calibrated before each visit by using a heat‐wire anemometer‐flowmeter *EasySPT200*. This Johnas 2 cascade impactor separates PM into three size fractions, PM_>10_, PM_2.5–10_ and PM_2.5_. Particles were impacted on electrostatic cloth,^46^ which was tested to release all allergens best (data not shown). PM was measured using a spectrometer *GRIMM model 1.108 version 8.60* in operational mode mass (normal dust mode, expressed in μg/m^3^), which divides PM into 16 fraction sizes every 6 s. We report PM as the same fractions as were collected with the Johnas 2, PM_2.5_, PM_2.5–10_ and PM_10–22.5_ by summing up the different Grimm channels accordingly. UFP (10–300 nm) were measured with a *Philips Aerosense Nano tracer* UFP and are reported as particles/cm^3^. Temperature, humidity and time were concomitantly measured with standard commercial sensors. Power consumption of air filtration (correlated with air flow) was monitored using a *Basetech EM‐3000*.

A 2‐min interval where pillows (30 s), covers (30 s) and sheets (60 s) were shaken represented one dust disturbance event. Each home visit consisted of four dust disturbance events. Air filtration at maximal performance (500 m^3^/h, lower flows can be easily set) was turned on directly after that event, and then the Johnas 2 was also turned on together with the spectrometer and the Nano tracer. After 1 h air filtration the devices were switched off and the procedure was repeated (each home was sampled about 4 h in total). The particle sensors ran continuously during the whole experiment, with doors and windows closed during the experiment. The home owners were told to not change the bedding within 2 weeks before each of the home visits and asked to not clean or vacuum the mattress in the bedroom until the end of (and 6 months before) the study. The same experimenter performed the dust disturbance events for control and intervention at a specific home. Nobody (including pets) was allowed to enter the experiment room or to take showers (humidity) during the experiment to avoid owners' interference in air quality. No home had multiple cats. All sampling inlets were located at 1.2 m' height and air filtration was located at least 2.5 m away from the measurement instruments. The only variation in experimental procedures between both visits was the in‐ or exclusion of the Philips air purifier. A drawing of the principal set‐up guaranteed that the equipment was placed identically for both visits (Figure S1).

### 2.5 Allergen Sampling

ELISA optimization for recovery of Der f 1 and Der p 1 was performed according the guidelines of EAACI.^47^ Here HDM allergens *D. pteronyssinus* Extract FD (Fetal Distress) excrements (Der f 1 < 0,05/Der p 2 = 0,78/Der p 1 = 22,3 mg/g), *D. pteronyssinus* Whole Cultures (Der f 1 = 0/Der p 2 = 0,64/Der p 1 = 6,74 mg/g), and *D. farinae* Whole Cultures (Der f 1 = 1,86/Der f 2 = 0,30/Der p 1 = 0 mg/g) from Cyteq Biologics, Groningen, Netherlands or EDC (Electrostatic dust collector)‐samples from homes were used.^46^, ^48^ All samples were extracted with 0.1 M ammonium bicarbonate pH = 8.0 containing 0.1% BSA, lyophilised, stored at −80°C and re‐suspended before analysis in 1/10 of the original volume in 0.1 M PBS pH = 7.2 + 0.05% Tween and 1% BSA. The limit of quantification (LOQ) in the calibration curve was 0.096 pg/m^3^ for Der f 1, 6.14 pg/m^3^ for Der p 1, 0.12 pg/m^3^ for Fel d 1 and 1.76 pg/m^3^ for Can f 1.

In the living‐rooms of four homes we mimicked standardized cleaning activities by deliberate resuspension of dust by brooming of the floor. The allergens were analysed using ELISAs for Der p 1, Der f 1, Fel d 1 and Can f 1 from Indoor Biotech, Charlottesvile USA with some modifications.^49^ Between the four repeats of each visit the filters were not changed resulting in a cumulative sample. This resulted in one allergen value per size fraction (six samples for each home in total: three samples per control‐ and three for intervention visit, one for each size fraction).

Homes with at least two values in any fraction >LOQ in control visits were included into analysis. Amongst the included homes, values below LOQ for any size fraction were set to half of LOQ.^50^, ^51^ This represents a conservative approach for statistical analysis since correctness of values below this LOQ can't be ensured. As a consequence, 20 homes qualified for analysis for Der f 1, 4 homes for Der p 1, 10 homes for Can f 1 and 21 homes for Fel d 1.

### 2.6 Data analysis

We checked the hypothesis whether air filtration after dust disturbance leads to a reduction in exposure to allergens and PM. Non‐normal distributions were found for allergens and PM by using a Shapiro–Wilk test. Thus, the Wilcoxon signed‐rank test was used to test our hypothesis. The only exceptions were those cases having *n* < 6 (Der p 1). Here, Wilcoxon cannot be used (*n* < 6) and a paired *t*‐test was used instead. Reduction (%) was calculated as = 100 − ((Intervention/Control) × 100).

For PM analysis, the area under the curve (AUC) was calculated by the trapezoid method for the times air filtration was running (2–60 min). In the PM analysis, the starting point of each repetition was defined as the first increase of the baseline^52^ and time was put to zero, except for UFP, where clock time was chosen instead since dust disturbances did not result in consistent peaks. In all cases (allergens and PM), statistical differences were reported when *p* < 0.05. To estimate the speed by which the air purifiers reach their maximum effect (i.e. after this time no more reduction in airborne particle reduction was measured) the average time until no more change in AUC is reached was calculated. All calculations were carried out by using R software^53^ and the AUC was calculated with the *pracma* package.^54^

## 3 RESULTS

### 3.1 Analysis for HDM allergens

Experiments in four homes showed lower Der f 1 concentrations (median = 63.2% less) in living rooms compared to bedrooms (Figure S2, Supplementary material). Hence, to avoid dilution of the pooled samples by low HDM allergen‐containing particles from the living room, we restricted our experimentation to the bedrooms.

The ELISA for Der f 1 was the most sensitive of our assays. We therefore used Der f 1 as a marker for HDM exposure. The Der p 1‐ELISA was sensitive enough to be able to detect allergen levels if they would have occurred at the same level as measured by the Der f 1 assay, but Der p 1 was detected only in four homes. The Der p 2 and Der f 2 assays were tested with pure HDM preparations from commercial suppliers and had a similar LOQ to the Der p 1 ELISA (data not shown), but did not detect sufficient allergen in pre‐experiment samples from homes. This indicates that they are infrequent allergens in and around Munich, and were omitted from the further study. Der f 1, in terms of positive homes, was the dominant allergen in Munich, Germany (Figure 1A,B).

FIGURE 1(A) Airborne concentrations of Der f 1 (*n* = 20). Left: Total airborne allergen (sum of all size fractions). Right: Concentrations for each size fraction. Dashed red lines represent the LOQ, which for Der f 1 is close to zero. Empty homes mean that they did not meet the criteria to be analyzed, according to section Allergen Sampling. (B) Airborne concentrations of Der p 1 (*n* = 4). Left: Total airborne allergen (sum of all size fractions). Right: Concentrations for each size fraction. Dashed red lines represent the LOQ. Empty homes mean that they did not meet the criteria to be analyzed, according to section Allergen Sampling

**Figure:**
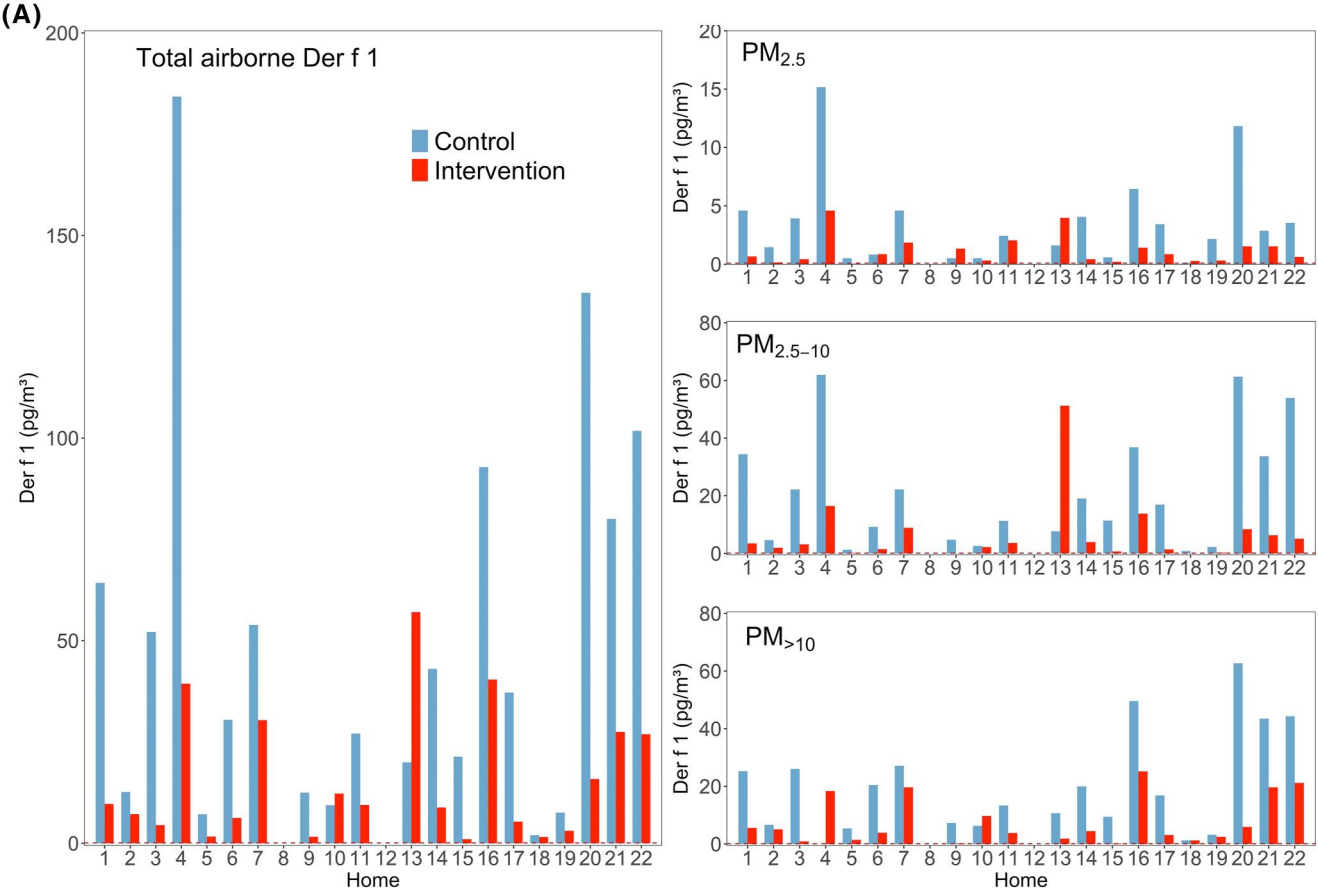

**Figure:**
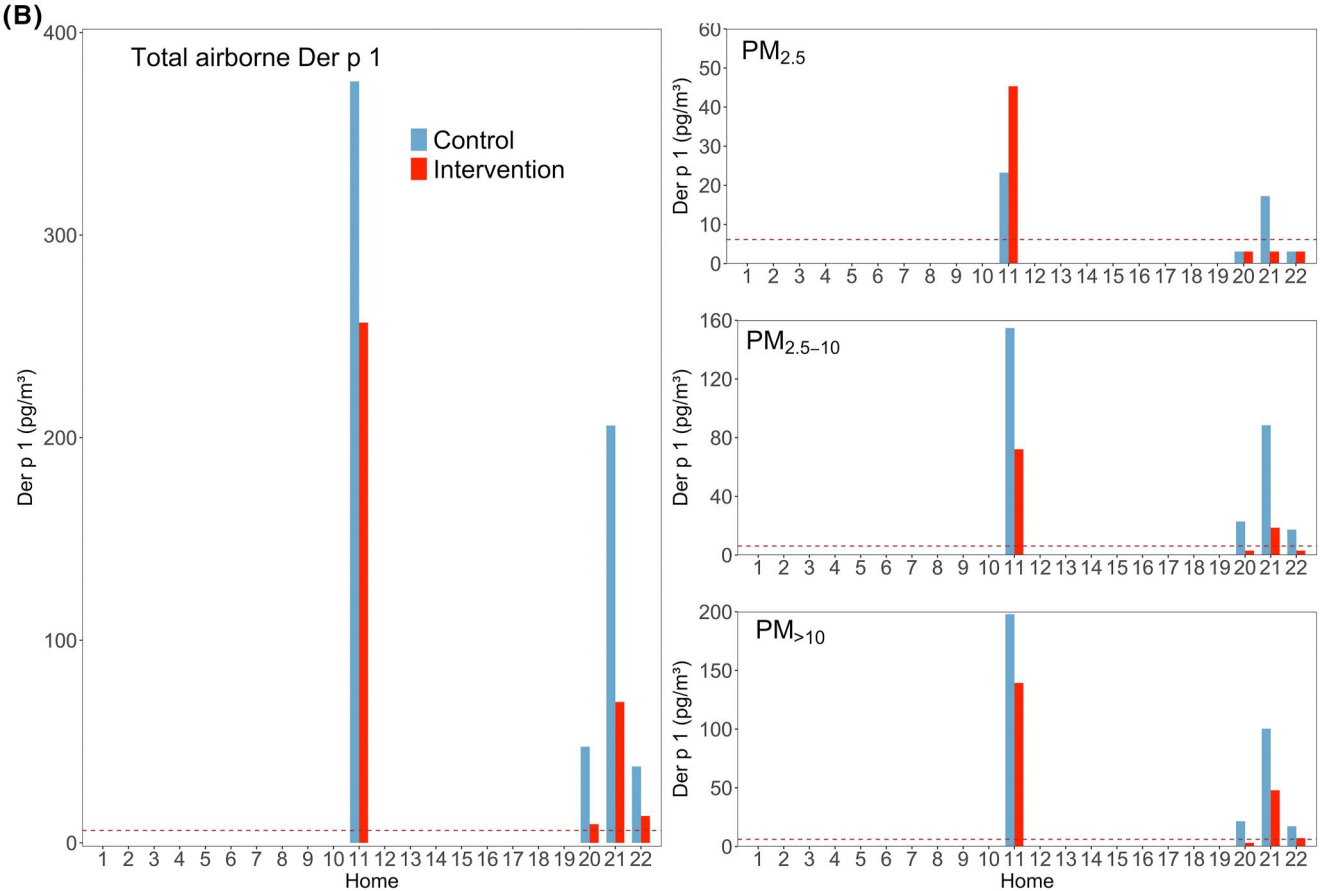

**FIGURE 2:**
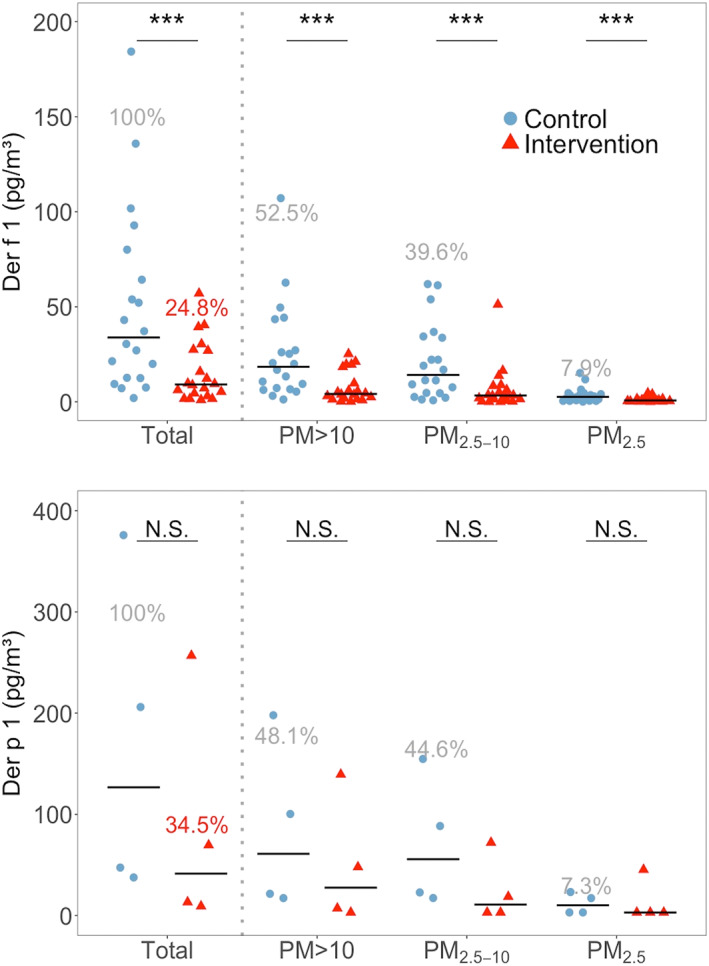
Total airborne mite concentrations of Der f 1 (upper) and Der p 1 (lower) and each size fraction during control and intervention visits. Horizontal black bars represent the medians. N.S, *, ** and *** represent *p* > 0.05, ≤0.05, ≤0.01 and ≤0.001, respectively. Percentages in grey show averaged allergen distribution across the three size fractions regarding the correspondent allergen sum. Percentages in red show the remaining allergen after intervention

Air filtration resulted in a statistically significant reduction in total airborne Der f 1 (*p* < 0.001; *n* = 20), while Der p 1 reduction failed to reach statistical significance (*p* = 0.066; *n* = 4). The median of allergen reduction was 75.2% and 65.5% for total Der f 1 and total Der p 1, respectively (Table 1). The reduction was quite homogeneous across all size fractions for Der f 1, whilst less consistent for Der p 1. The effect of air filtration was statistically significant in all the fractions for Der f 1 (*p* < 0.001), but was not significant for the Der p 1 fractions (*p* > 0.05; Figure 2) .

**TABLE 1:** Summary of reduction for all allergens in ambient air by air filtrations (expressed in %)

Home	Der f 1	Der p 1	Can f 1	Fel d 1	
Home 1	84.9	–	–	92.0	
Home 2	43.2	–	–	94.2	
Home 3	91.4	–	–	89.7	
Home 4	78.6	–	–	15.3	
Home 5	76.8	–	91.1	−225.3	
Home 6	79.5	–	89.0	89.5	
Home 7	43.6	–	47.8	93.0	
Home 8	–	–	98.4	96.1	
Home 9	87.0	–	–	84.9	
Home 10	−30.5	–	86.3	30.5	
Home 11	65.0	31.7	–	76.6	
Home 12	–	–	–	71.9	
Home 13	−185.6	–	–	68.3	
Home 14	79.5	–	93.9	66.1	
Home 15	95.2	–	–	77.6	
Home 16	56.5	–	–	−31.9	
Home 17	85.6	–	95.6	89.7	
Home 18	21.1	–	77.5	–	
Home 19	59.4	–	–	71.3	
Home 20	88.3	80.6	54.2	−91.8	
Home 21	65.7	66.2	89.6	85.8	
Home 22	73.5	64.8	–	45.7	
Median (*p*‐value)	75.2	65.5	89.3	76.6
	*p* < 0.001	*p* = 0.066	*p* < 0.01	*p* < 0.01

*Note*: Results expressed in median with *p*‐value included. Negative values represent those homes with non‐effective reduction.

The majority of Der f 1 and Der p 1 was detected in the fractions PM_>10_ and PM_2.5‐10_ (Figure 2). The descriptive statistics for HDM allergens can be seen in supplementary material (Table S2).

### 3.2 Analysis for cat and dog allergens

**FIGURE 4:**
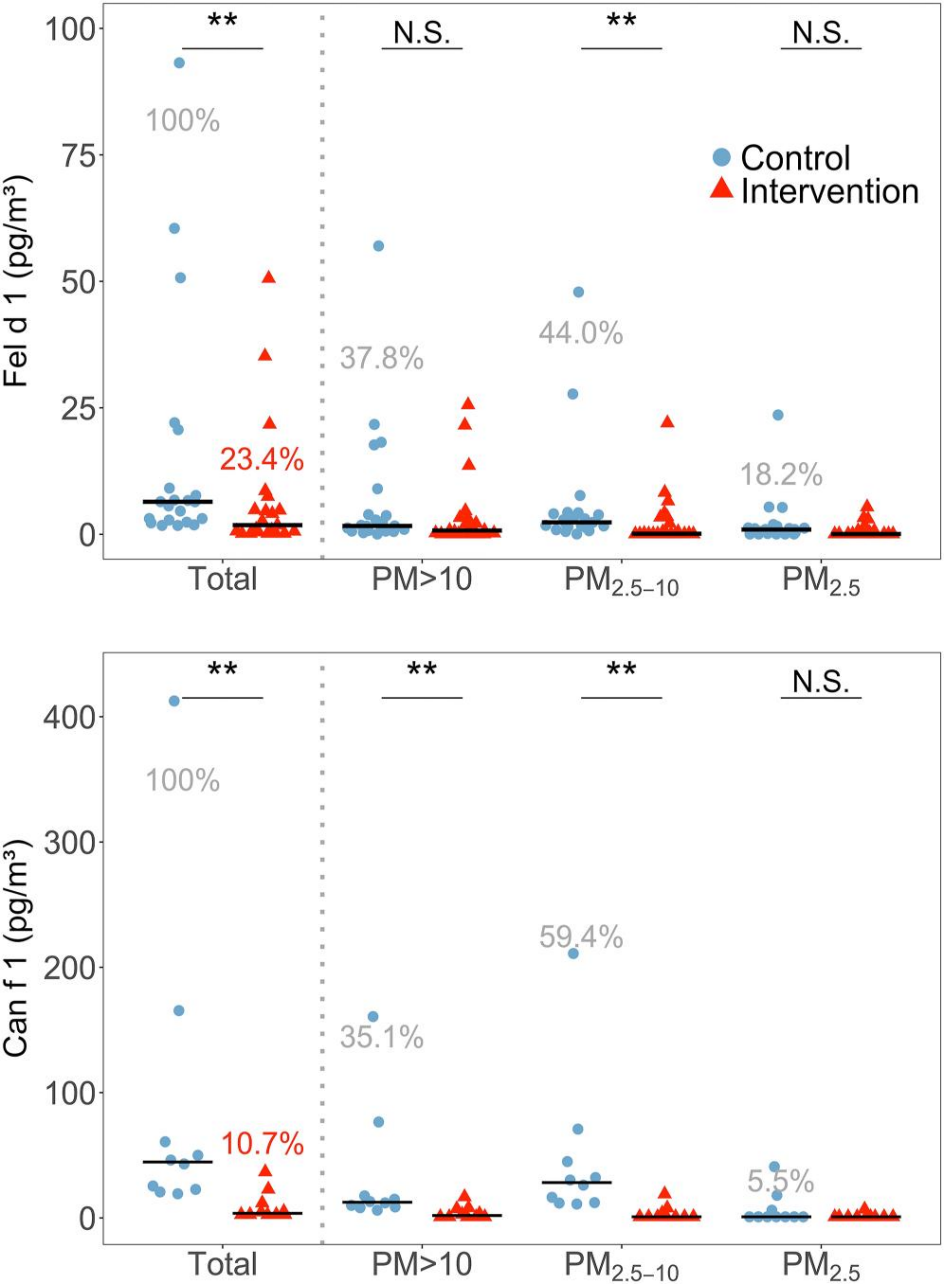
Total airborne concentrations of Fel d 1 (upper) and Can f 1 (lower) and each size fraction, during control and intervention visits. Horizontal black bars represent the medians. N.S, *, ** and *** represent *p*‐values > 0.05, ≤0.05, ≤0.01 and ≤0.001, respectively. Percentages in grey show averaged allergen distribution across the three size fractions regarding the correspondent allergen sum. Percentages in red show the remaining allergen after intervention

Measured Fel d 1 (cat) and Can f 1 (dog) allergen concentrations in each home are presented in Figure 3. Fel d 1 was detected in 21 homes during control visits, despite having a cat in three homes only. The dog allergen Can f 1 was detected in 10 homes, and here only two homes had a dog. Most allergen was associated with PM_2.5‐10_ and PM_>10_ as shown in Figure 4. Can f 1 concentrations in the two homes with the highest levels of this allergen were two and four times higher than the maximum for Fel d 1 (Figure 3A,B).

FIGURE 3(A) Airborne concentrations of Can f 1 (dog allergen), control and Intervention visits (*n* = 10). Left: Total airborne allergen (sum of all size fractions). Right: Concentrations for each size fraction. Dashed red lines represent the LOQs. Dashed black rectangles show the presence of a dog in homes. Empty homes mean that they did not meet the criteria to be analyzed, according to section Allergen Sampling. (B) Airborne concentrations of Fel d 1 (cat allergen), control and Intervention visits (*n* = 21). Left: Total airborne allergen (sum of all size fractions). Right: Concentrations for each size fraction. Dashed red lines represent the LOQs. Dashed black rectangles show the presence of a cat in homes. Empty homes mean that they did not meet the criteria to be analyzed, according to section Allergen Sampling

**Figure:**
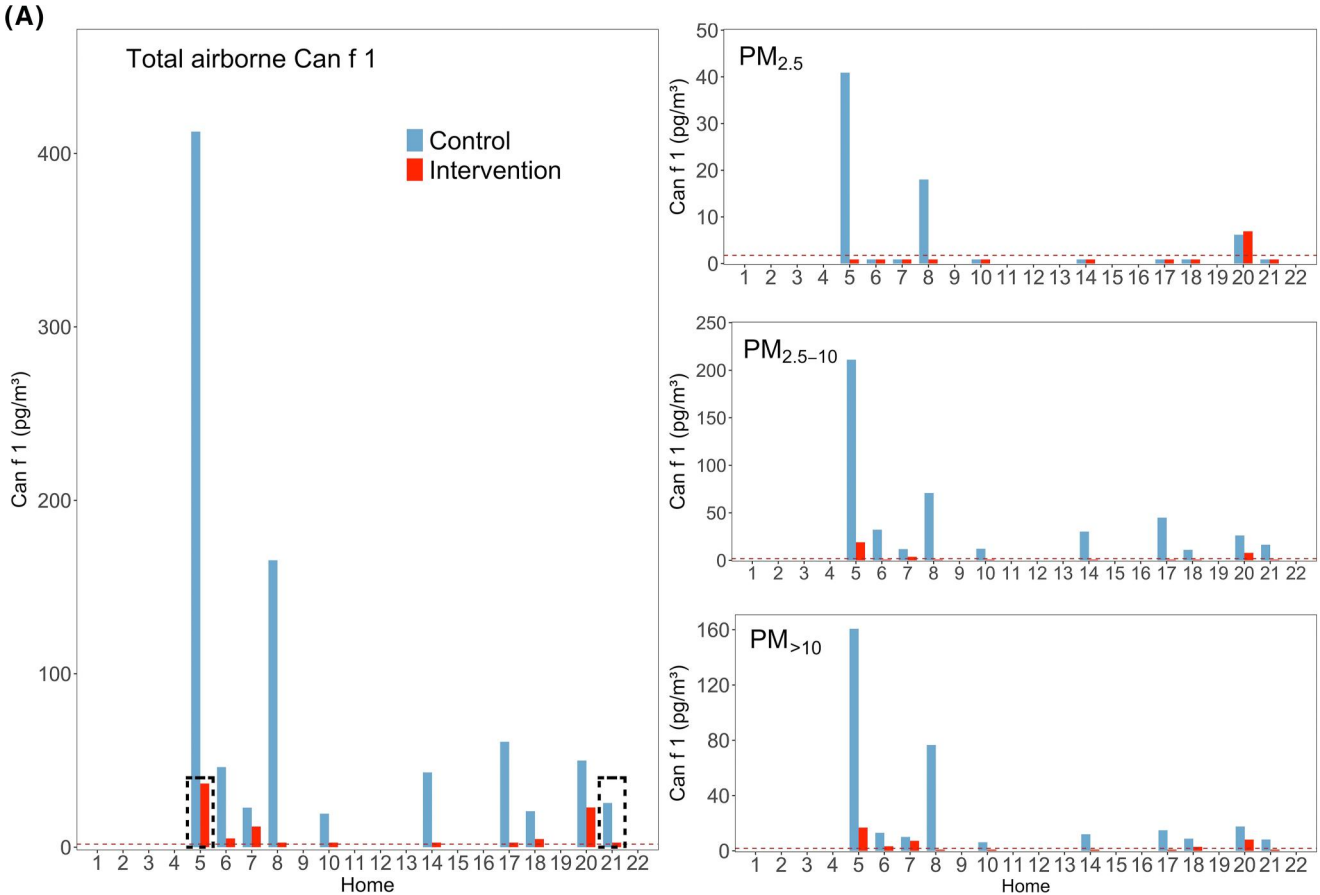

**Figure:**
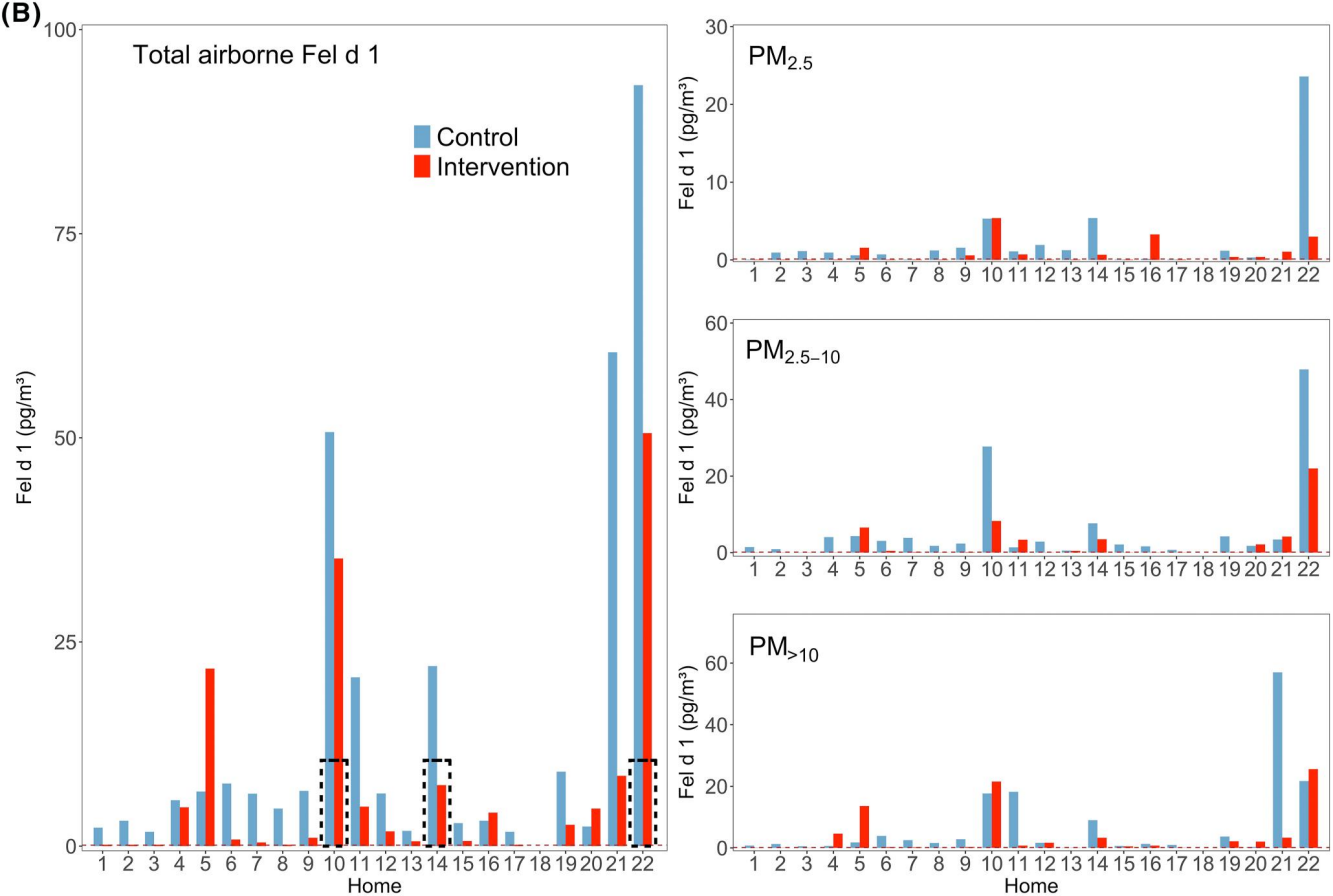

Air filtration resulted in a statistically significant reduction of total Fel d 1 (*p* < 0.01) and total Can f 1 (*p* < 0.01), with medians of reduction of 76.6% and 89.3%, respectively (Table 1). Fel d 1 was reduced significantly for PM_2.5‐10_ (*p* < 0.01; Figure 4). Can f 1 was reduced in all fractions that had a measurable concentration of allergen, obtaining medians in reductions of 87.5% for PM_>10_ (*p* < 0.01) and 93.7% for PM_2.5‐10_ (*p* < 0.01).

The median for Can f 1 for homes with a dog was 219.0 pg/m^3^ versus 22.8 pg/m^3^ in homes without a dog in control visits (*n* = 2 and *n* = 8), and 19.7 and 2.6 pg/m^3^ in homes with versus without a dog in intervention visits. For cats, the medians of Fel d 1 were 50.7 versus 5.1 pg/m^3^ for homes with and without a cat for control (*n* = 3 and *n* = 18), and 35.2 versus 0.9 pg/m^3^ for intervention. The descriptive statistics for cat and dog allergens can be seen in supplementary material (Table S2).

### 3.3 Analysis for particulate matter

**FIGURE 5:**
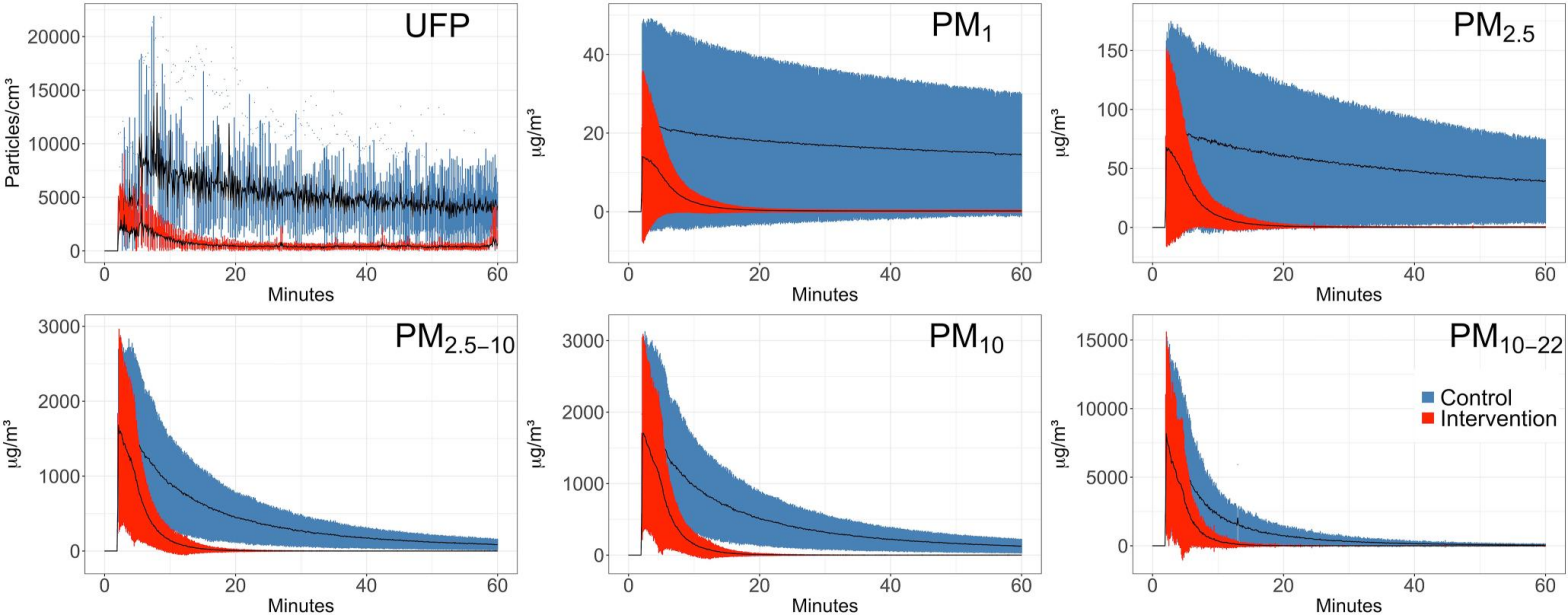
Change of PM in the bedrooms over the course of 1 hour following dust disturbance, with air filtration running (Intervention, red) versus without air purifier (Control, blue). Each curve represents the average of the four repetitions (all homes) for the different size fraction as indicated in each graph. Mean ± SD are given

Figure 5 shows the change of PM in the bedrooms over the course of 1 hour following dust disturbance (averages of 4 repeats and all homes). Dust disturbance at the start of each test resulted in instant and strong increases in airborne particles of all sizes, followed by continuous decline. The time after which no more particles are removed is 25 min for control and 10 min for intervention for PM_1_, for PM_2.5_ are 20 min (control) and 10 min (intervention), PM_2.5–10_ and PM_10_ are both 15 and 10 min, PM_10‐22.5_ are the same with 10 min for control and 10 min for intervention (data not shown). UFP shows an erratic behavior in time of removal, and results were inconsistent, probably due to the irregular peaks that this fraction presented during all the repetitions (outliers included). Please note that beside less time to reach no more reduction, also the concentration of final PM reached was always lower in intervention than with control.

The percentage of reduction as calculated using the AUCs for each home and fraction size and corresponding medians can be seen in Table 2. Running air filtration led to statistically significant reductions of PM concentrations in all particle sizes (all *p*‐values <0.001) with strongest effect for the medium size fractions.

**TABLE 2:** Summary for reduction for PM in ambient air by air filtration (expressed in %)

Home	UFP (10–300 nm)	PM_1_	PM_2.5_	PM_2.5–10_	PM_10_	PM_10–22_
Home 1	92.3	93.6	91.9	82.3	82.9	74.3
Home 2	78.9	92.4	90.2	81.3	82.2	66.9
Home 3	84.3	88.4	85.9	56.5	64.5	20.0
Home 4	79.1	91.1	89.1	74.8	74.2	64.4
Home 5	95.7	‐	‐	‐	‐	‐
Home 6	86.1	88.0	86.9	78.2	80.7	73.9
Home 7	92.3	93.3	91.9	70.3	72.9	36.6
Home 8	80.6	92.4	89.2	61.9	68.6	56.0
Home 9	88.8	91.9	90.8	84.2	84.2	77.0
Home 10	79.4	92.4	91.3	79.6	83.2	68.8
Home 11	89.3	89.7	89.2	81.4	82.6	72.1
Home 12	69.1	95.9	96.1	96.6	96.2	94.6
Home 13	83.3	81.1	77.1	30.1	33.7	−11.5
Home 14	82.6	95.6	92.9	75.1	78.5	70.8
Home 15	85.5	92.7	89.4	67.6	69.9	46.1
Home 16	85.3	94.1	93.8	90.0	90.0	90.4
Home 17	89.9	97.4	96.0	90.5	90.5	82.3
Home 18	92.9	87.8	85.1	49.8	51.5	7.8
Home 19	82.1	76.1	66.5	10.3	7.3	−18.6
Home 20	83.8	87.7	87.2	72.7	74.8	66.6
Home 21	83.8	93.3	92.6	78.1	79.6	67.2
Home 22	89.1	97.7	97.3	80.2	82.6	57.6
Median	92.3	93.6	91.9	82.3	82.9	74.3
*p*‐value	*p* < 0.001	*p* < 0.001	*p* < 0.001	*p* < 0.001	*p* < 0.001	*p* < 0.001

*Note*: Results expressed in median with *p*‐value included. Negative values represent those homes with non‐effective reduction.

## 4 DISCUSSION

Failure to measure any HDM allergen concentrations in homes is frequently reported,^13^, ^53^ as their concentrations are close to or below the limit of detection in many homes^54^, ^55^ or they deposit fast on the floor unless they were disturbed.^55^, ^56^, ^57^ For instance, a large study in U.S detected HDM allergens in only 38% of homes,^58^ and in Europe,^59^ detectable HDM allergens were reported for 49% of the samples. In the current study, the detection of airborne HDM allergen was successful in all homes even after splitting the allergens into three size fractions, due to optimization of the sampling, extraction and measurement protocols.^47^

Der f 1 was the dominant HDM allergen in Munich and surroundings (*n* = 20) and Der p 1 was only occasionally present (*n* = 4), but then at sometimes high levels (two sums for all fractions exceeded 200 pg/m^3^, Figure 1). The Der f 1 assay from Indoor Biotechnologies is more sensitive than the Der p 1 assay. However, the absence of Der p 1 in most homes was not due to the higher LOQ for Der p 1 compared to Der f 1, because if Der p 1 would have been present at the same concentrations as Der f 1, our assay would have easily detected it. In Bavaria, a previous study^60^ found that exposure to Der f 1, but not Der p 1, was associated with eczema and related to skin problems in 6‐7‐year‐old children. This, together with the higher abundance found in our study suggest that Der f 1 might be of higher clinical relevance in Bavaria compared to Der p 1.

*D. pteronyssinus* is more sensitive to desiccation under controlled laboratory conditions than *D. farinae*,^25^ which is consistent with Gross et al.,^51^ who reported higher Der p 1 concentrations with raising relative humidity and colder homes. These conditions were contrary to what we found in our homes (modern homes having better isolation and heating, which decreases humidity), which may explain the abundance of Der f 1 we observed in this study. Other authors also found a higher concentration of Der f 1 than Der p 1.^61^ Even in homes not infested by *D. farinae*, allergens can be transferred from mite‐infested clothing or car seat materials as a source of HDM allergens, as was reported by different authors.^61^, ^62^ Since outflow of the air purifier was vertical at 80 cm above the floor, and the inlet‐flow (0.9–1.1 m/sec) was close to the floor, but was filtered before emission, the air purifier itself could not cause resuspension of deposited allergens.

Air filtration resulted in a statistically significant reduction of HDM allergen Der f 1 but was not statistically significant for Der p 1 (see Figures 1 and 2). Hence, we think we missed statistical significance on Der p 1 due to too few homes containing that allergen in and around Munich.

Some authors reported 80% of HDM allergens to be associated with 10–40 μm particles,^11^, ^12^ settling down within 15 min and not remaining airborne due to rapid sedimentation.^63^ We also found most of Der f 1 and Der p 1 in the fraction PM_>10_, but our results showed that substantial allergen amounts stay airborne longer as half of total HDM allergen was carried by particles smaller than 10 microns. Furthermore, our Der f 1 (Figure 2) and PM (Figure 5 and Table 2) data show that despite faster sedimentation, significant exposure reduction can be achieved by means of air filtration even for large particles and associated allergens.

Some authors discussed that Fel d 1 is more suitable to be removed by air filtration, due to the higher percentage of allergen carried by smaller particles,^36^ which remain airborne over a longer period of time. Slower sedimentation times, at a given CADR mean higher removal contributions from air filtration versus those from natural decay, as observed in our study. We also found higher percentage of Fel d 1 and Can f 1to be carried by smaller (i.e. ≤ 10 microns) particles compared to mite allergens and a significant removal of total cat and dog allergen by air filtration. At the same time, these numbers show that around half of mite allergens can be carried by particles which remain airborne for significant periods of time. Furthermore, although our data confirm that a larger portion of pet allergen is associated with smaller particles (i.e. below 10 μm) compared to mite allergens, this difference was modest. Consequently, we were able to show that not only cat and dog allergens, but also those from mites can be significantly reduced by means of air filtration.

Our results are also in good alignment with previous findings from Custovic et al.,^64^, ^65^ who found 42% and 49% of total airborne Can f 1 and Fel d 1 to be associated with particles >9 μm. Luczynska et al.^66^ for Fel d 1 also reported a 75% association to particles larger than 5 μm. Tovey et al.^12^ reported that the 80% of Der p 1 was found in PM > 10 μm and De Blay et al.^63^ reported that 78% of the group I allergens of mite were detected in particles >6 μm. A direct comparison with our results is not possible as these studies used cascade impactors with different size fraction and methods of resuspension. Still, our study agrees with previous results that a large fraction of HDM was detected in the larger size fractions.^67^, ^68^, ^69^, ^70^ We show however that particles <10 µm also carry substantial amounts of HDM allergens.

Because airborne allergens like HDM are PM themselves, and air filtration used in this study was very effective in removing PM across a wide size range, removal of ambient PM also removed allergens. All PM fractions were significantly reduced by air filtration (all *p* < 0.001). The larger a particle, the faster it deposits by gravity and consequently air filtration has less time to ‘catch’ these particles. Consequently, small particles that stayed airborne longer like PM_1_ were more efficiently removed by air filtration than the larger particles like PM_10_ (Table 2).

Raulf et al.^47^ discussed that allergens on smaller particles remain airborne longer, and thus have more chance of being inhaled. Our time‐resolved PM measurements confirm this by showing a slower natural decay of smaller particles compared to larger ones (Figure 5). The same data also show, however, that all larger particles (except the largest size fraction PM_10–22.5_) remain elevated for at least 1 hour following dust disturbance. During this entire time these particles may be inhaled too. Hence, particles of all sizes should be removed as quickly as possible to minimize allergen exposure. In this study we demonstrated that all size fractions of PM were reduced to virtually zero in around 20 min with air filtration, impossible to achieve by relying on natural decay via sedimentation alone.

We found HDM allergens in all PM fractions of ambient air, with the majority carried by PM_>10_ followed by PM_2.5–10_. Can f 1 and Fel d 1 were also present in all PM fractions but most in the PM_2.5–10_ fraction (i.e. smaller than HDM). Although PM_2.5_ is very efficiently removed, allergen reduction in the corresponding size fraction did not reach statistical significance in any case except for Der f 1, likely because little allergen was found in this fraction.

Despite the use of HEPA air purifiers in previous studies to assess their effect on HDM,^39^, ^40^, ^50^ dog^48^, ^61^, ^62^, ^63^ and cat^34^, ^39^, ^48^, ^61^, ^62^, ^63^, ^64^ allergen exposure, these were either restricted to one^34^, ^39^, ^61^, ^64^ or two allergens,^37^, ^38^, ^62^, ^63^ based on one home only^50^ or did not measure airborne allergen levels.^37^, ^38^, ^62^, ^63^, ^64^ For instance, Jia‐Ying et al.^40^ showed a decrease in HDM allergens similar to the percentage reported in our study, but analyzing bedding and static dust samples (68.3% and 71.0%) and not airborne samples. The only study investigating the effect of portable HEPA air purifiers on airborne HDM allergen levels did not find a statistically significant effect.^39^ Stillerman et al.^71^ tested the effects of air filtration on mite, cat and dog allergens, but focused on the breathing zone and pillow encasements were also included, whilst Punsmann et al.^50^ studied dog, cat and mites allergens, but that study was limited to one home. A study with electrostatic air cleaners was carried out by Agrawal et al.,^72^ who reported a 60.3% reduction for Der f 1. However, they used cultures of *D. farinae* and the experiments were performed in a chamber, whilst our study was done in real bedrooms.

To our knowledge, the study presented here is the most extensive study focused exclusively on portable air filtration efficiency in bedrooms covering a wide range of airborne features: six fractions for PM, including UFP and four allergens. Air filtrations efficiently removed PM and consequently allergens from ambient air, which make them suitable to be added to the repertoire of allergen reducing measures. Air filtration removed finer PM more efficiently from the air than course particles because large particles by nature are also removed by sedimentation.

## 5 CONCLUSIONS

Airborne, inhalable HDM allergens limit patient well‐being already at low concentrations, that are difficult to measure. We were able, in contrast to other studies, due to the optimization of sampling, extraction and measuring protocols, to detect HDM allergen in all size fractions during household activities (changing the beddings). The major reduction for allergens were achieved in PM_2.5–10_ for HDM allergens (although HDM allergens were found in higher concentrations in PM_>10_) and also for Fel d 1 and Can f 1. Nevertheless, air filtration removed airborne particles from all size fractions ranging from 67% to 92.4% efficiency, and consequently removed particulate bound allergens like Der f 1 (75.2%; *p* < 0.001), Fel d 1 (76.6%; *p* < 0.01) and Can f 1 (89.3%; *p* < 0.01). Der p 1, probably due to the low (*n* = 4) number of houses where it was detected, was also reduced but the reduction missed statistical significance (65.5%; *p* = 0.066). We show that portable air filtration devices with an adequate CADR can be effective in reducing exposure to airborne allergens and particulate matter.

## CONFLICT OF INTEREST

Michael Scheja is employee in Philips. The investigations were carried out in compliance with good scientific practices. Support provided by Philips had no effect on the results presented.

## AUTHOR CONTRIBUTIONS

**Jose Maria Maya‐Manzano:** Data curation (equal); Formal analysis (lead); Methodology (equal); Software (lead); Validation (equal); Visualization (equal); Writing – original draft (lead); Writing – review & editing (equal). **Gudrun Pusch:** Data curation (equal); Formal analysis (equal); Investigation (equal); Methodology (supporting); Resources (equal); Writing – review & editing (supporting). **Cordula Ebner von Eschenbach:** Investigation (equal); Methodology (equal). **Elke Bartusel:** Investigation (equal); Methodology (equal). **Thomas Belzner:** Investigation (equal); Methodology (equal); Resources (equal). **Erwin Karg:** Conceptualization (equal); Investigation (equal); Methodology (equal); Writing – review & editing (equal). **Ulrich Bardolatzy:** Investigation (equal); Methodology (equal). **Michael Scheja:** Conceptualization (equal); Project administration (supporting); Resources (supporting); Software (supporting); Writing – review & editing (supporting). **Carsten Schmidt‐Weber:** Investigation (equal); Methodology (equal); Resources (equal), Writing – review & editing (equal). **Jeroen Buters:** Conceptualization (lead); Funding acquisition (lead); Investigation (lead); Methodology (equal); Project administration (lead); Resources (lead) Supervision (lead); Writing – original draft (supporting); Writing – review & editing (equal).

## Supporting information

Supplementary Material
