# Supplementary material for: Effect of air filtration on house dust mite, cat and dog allergens and particulate matter in homes
Source: Clin Transl Allergy. 2022 Apr 21;12(4):e12137. doi: 10.1002/clt2.12137 (PMC9022093; doi:10.1002/clt2.12137)

**Table S1.** Sampling period for each home.

| Home number | Date for Control | Date for Intervention |
| --- | --- | --- |
| 1 | 02/07/2020 | 30/07/2020 |
| 2 | 15/07/2020 | 11/08/2020 |
| 3 | 06/07/2020 | 03/08/2020 |
| 4 | 18/09/2020 | 21/08/2020 |
| 5 | 19/03/2020 | 05/02/2020 |
| 6 | 13/02/2020 | 12/03/2020 |
| 7 | 22/04/2020 | 25/03/2020 |
| 8 | 04/09/2020 | 12/08/2020 |
| 9 | 10/02/2020 | 10/03/2020 |
| 10 | 27/02/2020 | 30/01/2020 |
| 11 | 12/03/2020 | 13/02/2020 |
| 12 | 11/02/2020 | 09/03/2020 |
| 13 | 24/09/2020 | 27/08/2020 |
| 14 | 28/09/2020 | 01/09/2020 |
| 15 | 08/07/2020 | 05/08/2020 |
| 16 | 14/07/2020 | 06/08/2020 |
| 17 | 21/09/2020 | 24/08/2020 |
| 18 | 28/02/2020 | 31/01/2020 |
| 19 | 17/04/2020 | 16/03/2020 |
| 20 | 14/02/2020 | 20/03/2020 |
| 21 | 01/07/2020 | 29/07/2020 |
| 22 | 21/02/2020 | 23/03/2020 |

**Table S2.** Descriptive statistics for each fraction size (average values of allergen and confidence intervals 95%, pg/m^3^).

|  | Control | | | Intervention | | |
| --- | --- | --- | --- | --- | --- | --- |
|  | PM_>10_ | PM_10_ | PM_2.5_ | PM_>10_ | PM_10_ | PM_2.5_ |
| Der f 1  (n=20) | 25.3  [13.3, 37.4] | 20.9  [11.6, 30.2] | 3.6  [1.7, 5.4] | 7.7  [3.9, 11.5] | 6.6  [1.3, 12.0] | 1.2  [0.6, 1.7] |
| Der p 1  (n=4) | 84.3  [0.0, 219.0] | 70.8  [0.0, 174.0] | 11.6  [0.0, 27.9] | 49.4  [0.0, 150.0] | 24.2  [0.0, 76.3] | 13.6  [0.0, 47.2] |
| Can f 1  (n=10) | 32.8  [0.0, 68.2] | 46.7  [3.3, 90.1] | 7.1  [0.0, 16.5] | 4.3  [0.6, 8.0] | 3.7  [0.0, 7.9] | 1.5  [0.1, 2.8] |
| Fel d 1  (n=21) | 7.0  [1.1, 13.0] | 5.9  [0.8, 11.0] | 2.3  [0.0, 4.6] | 3.9  [0.6, 7.2] | 2.5  [0.2, 5.0] | 0.8  [0.2, 1.5] |

**Figure S1:** Experimental set up for each Dust disturbance Event and for the location for measure instruments within the bedrooms.

**
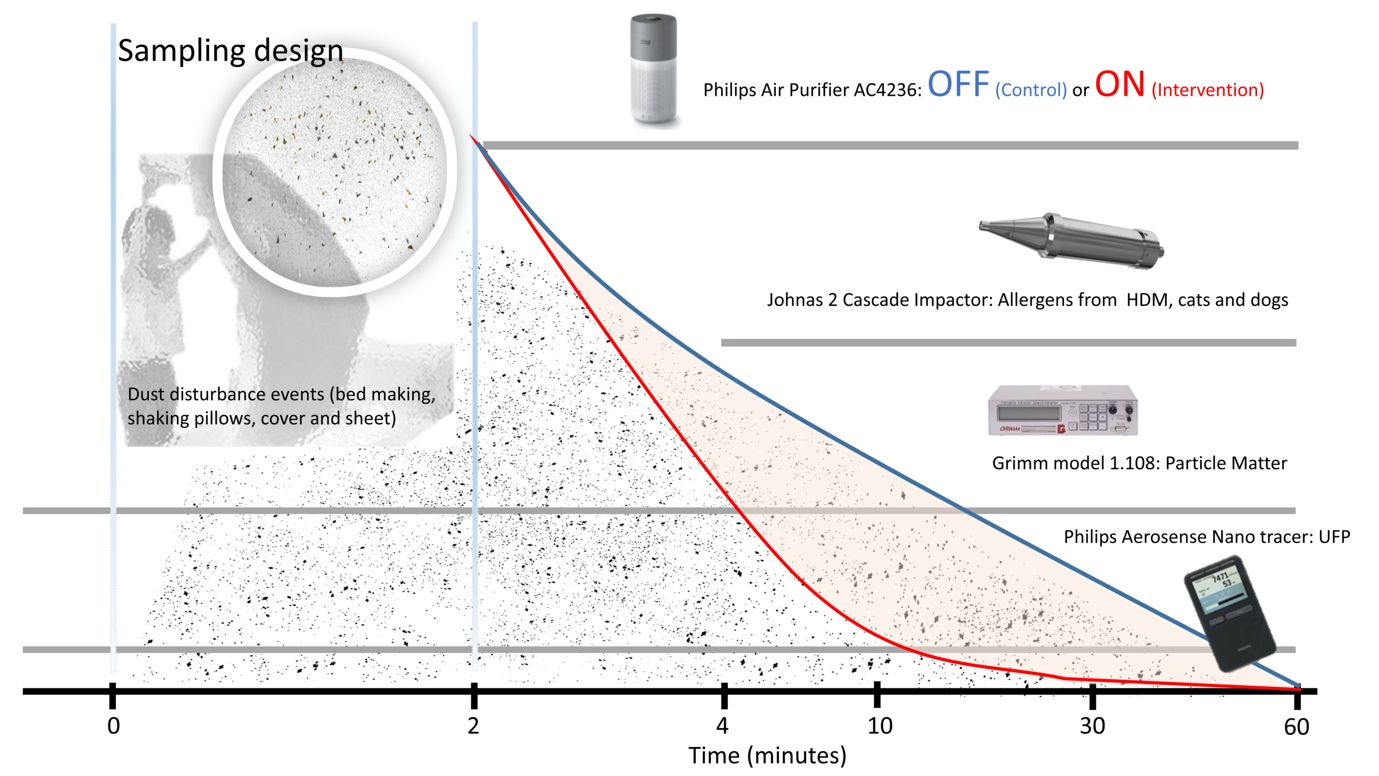
**

**
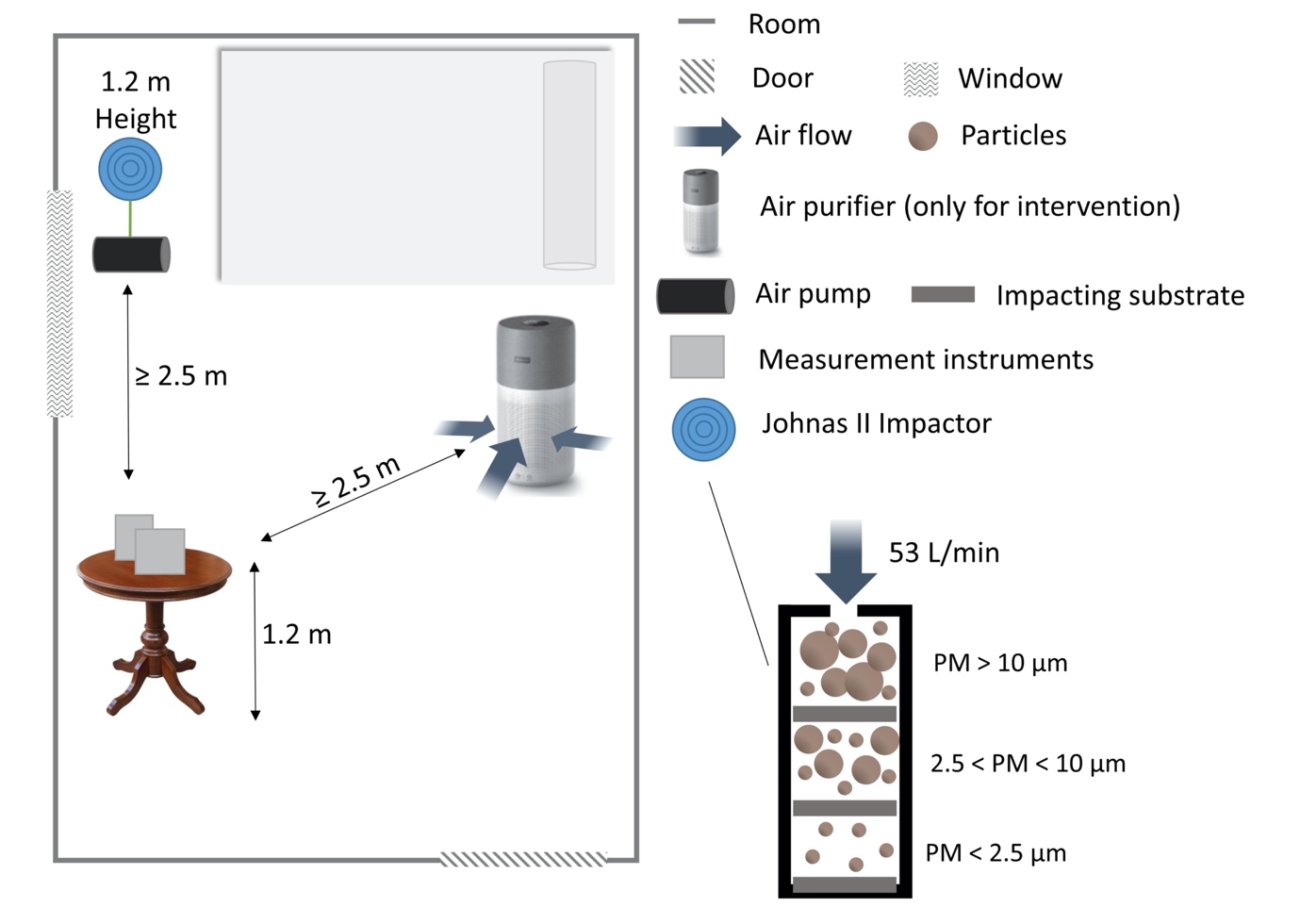
**

**Figure S2:** Der f 1 in ambient air in living rooms vs. bedrooms. The sum of Der f 1 in the different PM size fractions is given. The Bedrooms are shown in dark green and living rooms in orange. Subsequently only bedrooms were sampled.


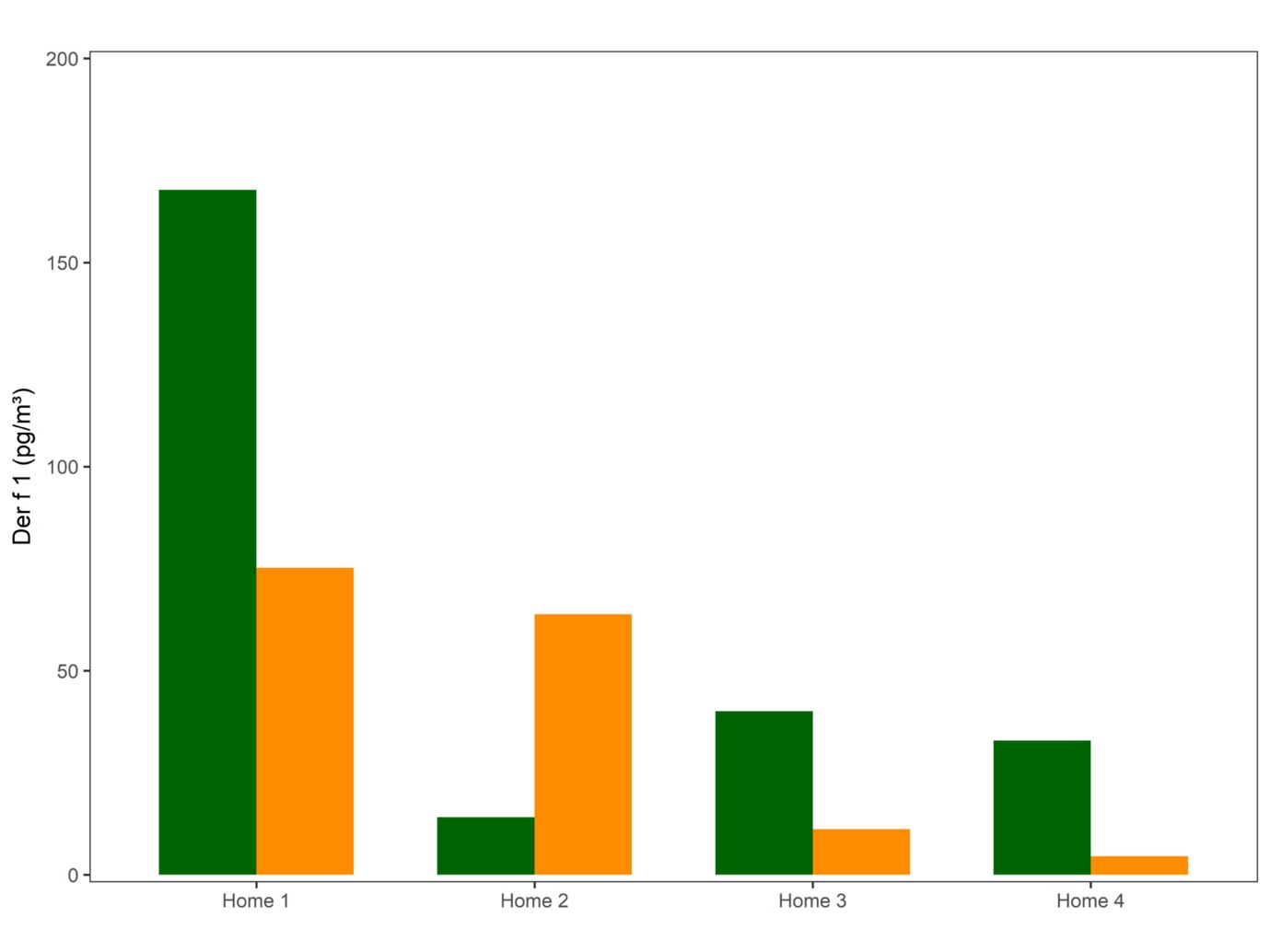

Supplement: Supplementary file 1 — Supplementary Material [file CLT2-12-e12137-s001.docx]
